# Association between vitiligo and smoking: A nationwide population-based study in Korea

**DOI:** 10.1038/s41598-020-63384-y

**Published:** 2020-04-10

**Authors:** Young Bok Lee, Ji Hyun Lee, Soo Young Lee, Dong Soo Yu, Kyung Do Han, Yong Gyu Park

**Affiliations:** 1 Department of Dermatology, College of Medicine, The Catholic University of Korea, Seoul, Korea; 2 Department of Biostatistics, College of Medicine, The Catholic University of Korea, Seoul, Korea

**Keywords:** Vitiligo, Risk factors

## Abstract

No study has examined the associations between vitiligo and smoking. The purpose of this study was to investigate the incidence of vitiligo according to smoking status. We used clinical data from individuals aged over 20 years who received a health examination in the National Insurance Program between 2009 and 2012 (n = 23,503,807). We excluded individuals with pre-existing vitiligo who had ever been diagnosed with vitiligo before the index year (n = 35,710) or who were diagnosed with vitiligo within a year of the index year (n = 46,476). Newly diagnosed vitiligo was identified using claims data from baseline to date of diagnosis or December 31, 2016 (n = 22,811). The development of vitiligo was compared according to self-reported smoking status by a health examination survey. The hazard ratio of vitiligo in current smokers was 0.69 (95% confidence interval; 0.65–0.72) with a reference of never-smokers after adjustment for age, sex, regular exercise, drinking status, body mass index, diabetes mellitus, hypertension, dyslipidemia, history of stroke, and history of ischemic heart diseases. The decreased risk of vitiligo in current smokers persisted after subgroup analysis of sex and age groups. The results suggested there are suppressive effects of smoking on the development of vitiligo. Further studies are needed to evaluate the mechanism of smoking on the development of vitiligo.

## Introduction

Vitiligo is a common depigmentation skin disease with an estimated prevalence of 0.5–1% in the worldwide population^1^. Vitiligo affects all skin types and ethnic groups. The highest incidence is recorded in India (up to 8.8%)^2^, followed by Mexico (4%)^3^, Japan (1.68%)^4^, and Denmark (0.38%)^5^. The annual incidence of vitiligo in South Korea was estimated at 0.12–0.13%^6^.

Vitiligo is a multifactorial disease. It is hypothesized to be mainly caused by autoimmune factors, although genetic susceptibility, oxidative stress, and cell detachment abnormalities are also suggested etiologies^7^. The autoimmune diseases vitiligo is associated with include autoimmune thyroid disorders, rheumatoid arthritis, adult-onset diabetes mellitus, pernicious anemia, and systemic lupus erythematosus^8,9^.

Once vitiligo develops, it is difficult to treat and affects quality of life. Vitiligo can negatively affect social relations^10–13^. One study showed that vitiligo has an adverse effect on patient sexuality^14^, and it has been associated with pessimistic emotions such as shame, insecurity, and sadness^15^. Therefore, evaluating the preventive and risk factors for vitiligo is important.

Smoking is one of the most prevalent addictive habits that affects multi-organ systems and results in several diseases. The well-known risks of smoking habits include respiratory and cardiovascular diseases. Smoking also affects the immune system and results in inflammatory reactions. Tobacco contains as many as 6,000 different components including nicotine, polycyclic aromatic hydrocarbons, tobacco glycoprotein, and some metals, many of which are considered antigenic, cytotoxic, mutagenic, and carcinogenic. The effects of smoking are considered to be harmful for human health. In terms of autoimmune skin diseases, the results have been conflicting according to various skin diseases. Smoking is known to have detrimental effects on and a positive association with psoriasis^16–19^, palmoplantar pustulosis,^20^ and hidradenitis suppurativa^21–27^. However, several autoimmune skin diseases, such as pemphigus vulgaris, foliaceous, and^28–33^ Behçet’s disease^34–36^, show a negative association with smoking. We recently reported that the incidence of Behçet’s disease in current smokers was significantly decreased compared to that of never-smokers in South Korea^36^. Until now, the association between vitiligo and smoking has not been evaluated or reported.

Herein, this study aimed to investigate the incidence of vitiligo according to smoking habits using a nationwide population-based cohort design to analyze data from the National Health Insurance Research Database in Korea.

## Materials and Methods

### Study design and database

This study used the same dataset that was previously reported;^36^ the Korean Health Examination database and the Korean National Health Insurance Service (KNHIS) claims database. Briefly, the KNHIS database contains all claims data for the KNHIS program, the Korean Medical Aid program, and all other long-term care insurance programs for 99% of the Korean population and the Korean National Health Examination database was used to select participants and obtain information on confounding variables. The health examination data included anthropometric measures, smoking status, drinking, and exercise, and self-reported medical histories. Per the methods in the previous paper^36^, we used the linked KNHIS claims database for the same individuals to evaluate the development of vitiligo.

This nationwide, population-based retrospective cohort study used the KNHIS Claims Database (diagnoses according to the International Classification of Disease, Tenth Revision [ICD-10] code). The Institutional Review Board at the Korea Centers for Disease Control and Prevention approved the protocol (NHIS-2018-1-333). The study was also approved by the Institutional Review Board at Uijeongbu St. Mary’s Hospital, Catholic University of Korea and was conducted according to the principles of the Declaration of Helsinki. Anonymized and de-identified information were used for analyses, and therefore informed consent was not required.

### Study population

**Figure 1:**
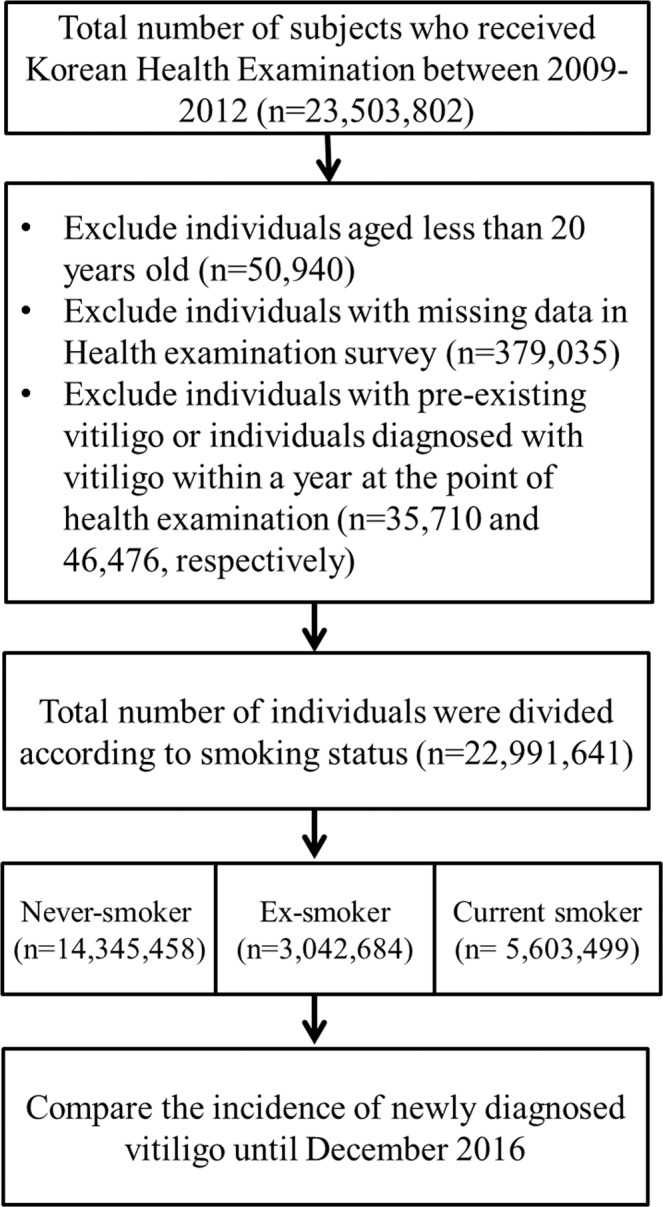
Study design flow chart.

The total number of individuals who underwent National Health Examinations in Korea from 2009 until 2012 was 23,503,802. The first year an individual received a health examination was considered the index year. We excluded individuals aged less than 20 years (n = 50,940) or who had missing data in the health examination survey (n = 379,035). To identify newly diagnosed vitiligo, we excluded individuals with pre-existing vitiligo who had ever been diagnosed with vitiligo before the index year (n = 35,710) or who were diagnosed with vitiligo within a year of the index year (n = 46,476). The observation began a year after the date of the baseline Health Examination in all subjects and ended when vitiligo was diagnosed or December 31, 2016. (Fig. 1)

### Subgroups according to smoking status

Smoking status was obtained from a self-reported questionnaire during the health examination. Study individuals (n = 22,991,641) were divided into three groups according to smoking status; never-smoker (n = 14,345,458), ex-smoker (n = 3,042,684), and current smoker (n = 5,603,499). (Fig. 1) The amount of smoking was sub-grouped into <10 cigarettes per day, 10–19 cigarettes per day, and ≥20 cigarettes per day, and smoking duration was divided into <10 years, 10–29 years, and ≥30 years.

### Comorbidities

To adjust for comorbidities, the presence of diabetes mellitus, hypertension, and dyslipidemia were defined using ICD-10 codes: diabetes mellitus (E11-14), hypertension (I10-13 and I15), and dyslipidemia (E78) with medication in KNHIS database. History of stoke or ischemic heart disease was obtained by self-reported questionnaire during the National Health examination.

### Statistical analysis

We considered comorbidities including diabetes mellitus, hypertension, dyslipidemia, ischemic heart disease, and stroke as possible confounders to adjust in our analyses. Cox’s proportional hazard regression models with an age timescale were used to identify the associations between smoking status and newly diagnosed vitiligo. Hazard ratio (HR) and 95% confidence intervals (CI) for smoking status were compared to the reference (never-smoker). We performed the subgroup analyses separately for men and women and age groups (20–39 years, 40–65 years, ≥65 years). Proportional hazard assumptions were checked using log-log cumulative survival graphs and the time-dependent variable Cox model after adjustment for baseline covariates including age, sex, regular exercise, drinking status, BMI, diabetes mellitus, hypertension, dyslipidemia, history of stroke, and history of myocardial infarction, according to smoking status. All statistical analyses were performed using SAS software (ver. 9.4; SAS Institute, Cary, NC, USA).

## Results

### Characteristics of the study population

Among the 22,991,641 individuals, 14,345,458 were never-smokers, 3,042,684 were ex-smokers, and 5,603,499 were current smokers. The current smokers (43.34 ± 12.87 years) were younger than never-smokers (48.86 ± 14.8 years) and ex-smokers (50.11 ± 13.4 years). The percentage of men was significantly higher in ex-smokers and current smokers than in never-smokers. Drinking habits (2–3 times a month) were higher in ex-smokers and current smokers. Comorbidities including diabetes mellitus, hypertension, and dyslipidemia were highest in ex-smokers. History of previous stroke and ischemic heart disease was also higher in ex-smokers. (Table 1).

**Table 1:** Demographics of study population.

	Never-smoker	Ex-smoker	Current smoker	
N	14,345,458	3,042,684	5,603,499	P value
Mean age (years)	48.86 ± 14.8	50.11 ± 13.35	43.34 ± 12.87	<0.001
**Age group**				
20–39	3,684,294(25.68)	672,772(22.11)	2,321,427(41.43)	
40–64	8,309,359(57.92)	1,903,257(62.55)	2,898,057(51.72)	
≥65	2,351,805(16.39)	466,655(15.34)	384,015(6.85)	
Male (%)	3,615,580(25.2)	2,830,044(93.01)	5,149,573(91.9)	<0.0001
Urban habitat (%)	6,587,708(45.95)	1,443,414(47.46)	2,498,188(44.61)	<0.0001
Drinking status (2–3 times a month)	270,927(1.89)	355,742(11.69)	885,652(15.81)	<0.0001
Regular exercise (more than 3 times per a week, %)	6,565,007(45.76)	1,890,240(62.12)	2,936,635(52.41)	<0.0001
BMI (kg/cm^2^)	23.46 ± 3.31	24.36 ± 2.96	23.91 ± 3.28	<0.0001
<18.5	677,515(4.72)	58,319(1.92)	194,862(3.48)	
<23	6,128,109(42.72)	924,509(30.38)	2,093,363(37.36)	
<25	3,332,620(23.23)	85,0225(27.94)	1,375,213(24.54)	
<30	3,697,465(25.77)	1,100,683(36.17)	1,705,002(30.43)	
≥30	509,749(3.55)	108,948(3.58)	235,059(4.19)	
Height (cm)	159.75 ± 8.41	168.74 ± 6.81	169.68 ± 7.24	<0.0001
Weight (kg)	60 ± 10.52	69.52 ± 10.43	69.06 ± 11.59	<0.0001
Waist circumference (cm)	78.14 ± 9.27	84.16 ± 8.07	82.59 ± 8.49	<0.0001
Diabetes	1,217,006(8.48)	377,630(12.41)	525,950(9.39)	<0.0001
Glucose (mmol/L)	96.35 ± 21.85	101.02 ± 25.14	98.63 ± 25.78	<0.0001
Hypertension	3,757,617(26.19)	1,022,615(33.61)	1,271,606(22.69)	<0.0001
Diastolic blood pressure(mmHg)	75.14 ± 10.12	78.12 ± 9.93	77.31 ± 9.85	<0.0001
Systolic blood pressure(mmHg)	121.15 ± 15.59	125.22 ± 14.54	123.36 ± 14.19	<0.0001
Dyslipidemia	2,836,746(19.77)	664,482(21.84)	932,288(16.64)	<0.0001
Cholesterol(mmol/L)	194.7 ± 36.97	195.53 ± 36.56	194.26 ± 36.81	<0.0001
Previous stroke (%)	130,454(1.43)	52,808(2.57)	28,561(0.8)	<0.0001
Previous myocardial infarction (%)	294,495(3.21)	103,386(5.03)	62,761(1.76)	<0.0001

### Newly diagnosed vitiligo according to smoking status

**Figure 2:**
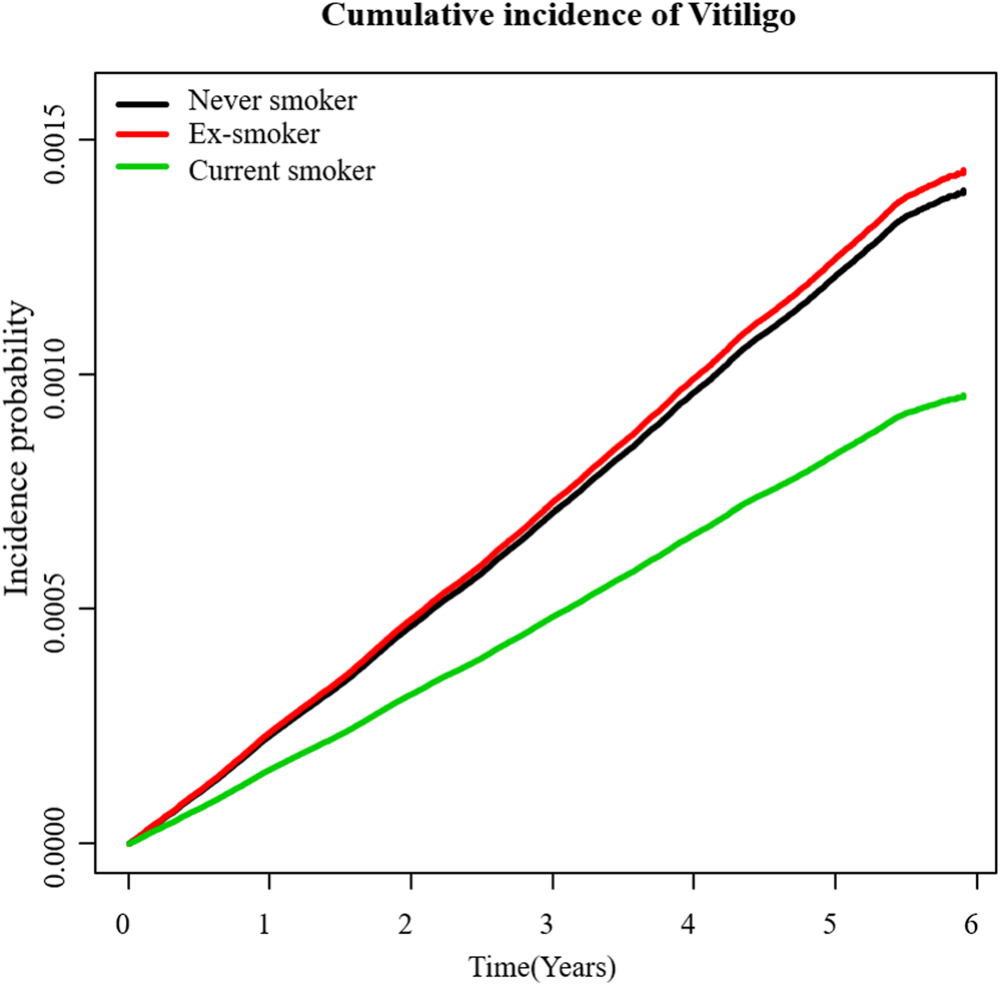
An adjusted cumulative incidence of vitiligo for baseline covariates including age, sex, regular exercise, drinking status, BMI, diabetes mellitus, hypertension, dyslipidemia, history of stroke, and history of myocardial infarction, according to smoking status.

There were 16,515 newly diagnosed cases of vitiligo in never-smokers, 3,003 cases in ex-smokers, and 3,293 cases in current smokers. The incidence of newly diagnosed vitiligo was 2.63 per 10,000 person-years in never-smokers, 2.23 per 10,000 person-years in ex-smokers, and 1.35 per 10,000 person-years in current smokers. The cumulative incidence of newly diagnosed vitiligo after adjustment for covariates is shown in Fig. 2 according to smoking status. Current smokers had a significantly lower risk of vitiligo (HR 0.51, 95% CI, 0.50–0.53) compared with never-smokers. Decreased risk of newly diagnosed vitiligo in current smokers persisted after setting an age timescale in Model 1 (HR 0.56, 95% CI, 0.54–0.59) and adjustment for covariates in Model 2 (HR 0.68, 95% CI, 0.64–0.71) and Model 3 (HR 0.69, 95% CI, 0.65–0.72). Sensitivity analysis also showed decreased vitiligo risk in current smokers regardless of age or sex (Table 2).

**Table 2:** Incidence rates of vitiligo according to smoking status.

Subgroup	Smoking status	Number	Vitiligo	Person-years	Incidence rate (per 10,000 person-years)	HR(95%C.I)		
						Model 1	Model 2	Model 3
Total	Non	14,345,458	16,515	62,819,781.13	2.63	1(ref.)	1(ref.)	1(ref.)
	Ex	3,042,684	3,003	13,438,871.66	2.23	0.83(0.79–0.86)	1(0.95–1.05)	1(0.95–1.05)
	Current	5,603,499	3,293	24,374,473.09	1.35	0.56(0.54–0.59)	0.68(0.64–0.71)	0.69(0.65–0.72)
Male	Non	3,615,580	3,399	16,007,707.47	2.12	1(ref.)	1(ref.)	1(ref.)
	Ex	2,830,044	2,781	12,572,432.85	2.21	1.01(0.95–1.06)	1(0.95–1.06)	1(0.95–1.06)
	Current	5,149,573	2,905	22,525,419.59	1.29	0.65(0.62–0.69)	0.66(0.62–0.69)	0.67(0.63–0.71)
Female	Non	10,729,878	13,116	46,812,073.66	2.80	1(ref.)	1(ref.)	1(ref.)
	Ex	212,640	222	866,438.81	2.56	1.03(0.89–1.19)	1.03(0.89–1.18)	1.03(0.89–1.18)
	Current	453,926	388	1,849,053.5	2.10	0.79(0.71–0.88)	0.79(0.71–0.88)	0.79(0.7–0.88)
20–39	Non	3,684,294	2,860	15,877,168.48	1.80	1(ref.)	1(ref.)	1(ref.)
	Ex	672,772	491	3,017,749.1	1.63	0.9(0.81–1)	0.95(0.85–1.06)	0.96(0.86–1.08)
	Current	2,321,427	1,170	10,239,014.55	1.14	0.63(0.59–0.68)	0.68(0.62–0.74)	0.69(0.63–0.75)
40–64	Non	8,309,359	10,707	36,527,675.57	2.93	1(ref.)	1(ref.)	1(ref.)
	Ex	1,903,257	1,952	8,411,829.96	2.32	0.78(0.74–0.82)	1.01(0.94–1.07)	1.01(0.95–1.08)
	Current	2,898,057	1,818	12,483,012.69	1.46	0.52(0.5–0.55)	0.67(0.63–0.71)	0.67(0.63–0.71)
65-	Non	2,351,805	2,948	10,414,937.08	2.83	1(ref.)	1(ref.)	1(ref.)
	Ex	466,655	560	2,009,292.61	2.79	0.96(0.87–1.05)	1.1(0.98–1.23)	1.1(0.98–1.24)
	Current	384,015	305	1,652,445.85	1.85	0.63(0.56–0.71)	0.71(0.62–0.82)	0.75(0.65–0.86)

Model 1 Not adjusted with setting an age timescale

Model 2 Adjusted by baseline age and sex with setting an age timescale.

Model 3 Adjusted by baseline age, sex, regular exercise, drinking status, BMI, diabetes mellitus, hypertension, dyslipidemia, history of stroke, and history of myocardial infarction with setting an age timescale.

Hazard ratios for vitiligo development in ex-smokers and current smokers were obtained with reference to never-smokers. An age timescale was used in Model 1, 2, and 3. Subgroup analyses were evaluated by sex and age group.

### Subgroup analysis of amount and duration of smoking

Current smokers showed a decrease in HR for newly diagnosed vitiligo in proportion to the amount of smoking compared with never-smokers; HR 0.70, 95% CI, 0.64–0.76) in current smokers who smoked less than 10 cigarettes a day, HR 0.51, 95% CI, 0.49–0.52 in current smoker who smoked 10–29 cigarettes a day, and HR 0.47, 95% CI, 0.44–0.49 in current smoker who smoked more than 30 cigarettes a day. (Table 3) Current smokers showed decreased HR for vitiligo regardless of smoking duration compared with never-smokers.

**Table 3:** Hazard ratios of vitiligo development in association with amount and duration of smoking.

	Number	Vitiligo	Person-years	Incidence rate (per 10,000 person-years)	HR(95%C.I)		
					Model1	Model2	Model3
**Amount of smoking**							
Never-smoker	14,345,458	16,515	62,819,781.1	2.63	1(ref.)	1(ref.)	1(ref.)
Ex-smoker							
smoked <10 cigarettes/day	475,960	472	2,056,674.1	2.29	0.87(0.80,0.96)	1.04(0.95,1.14)	1.03(0.94,1.13)
smoked 10–19 cigarettes/day	1,160,814	1,124	5,173,202.4	2.17	0.83(0.78,0.88)	1.01(0.95,1.08)	1.01(0.95,1.1)
smoked ≥20 cigarettes/day	1,405,910	1,407	6,208,995.1	2.27	0.86(0.82,0.91)	1.01(0.95,1.08)	1.03(0.97,1.10)
Current smoker							
smokes <10 cigarettes/day	692,322	538	2,933,516.2	1.83	0.70(0.64,0.76)	0.85(0.78,0.93)	0.86(0.79,0.94)
smokes 10~19 cigarettes/day	2,371,434	1,396	10,335,923.5	1.35	0.51(0.49,0.54)	0.69(0.65,0.73)	0.69(0.65,0.74)
smokes ≥20 cigarettes/day	2,539,743	1,359	11,105,033.4	1.22	0.47(0.44,0.49)	0.60(0.56,0.64)	0.62(0.58,0.66)
**Duration of smoking**							
Never-smoker	14,345,458	16,515	62,819,781.1	2.63	1(ref.)	1(ref.)	1(ref.)
Ex-smoker							
smoked for <10 years	708,297	635	3,093,384.6	2.04	0.78(0.72,0.84)	1.02(0.94,1.11)	1.02(0.94,1.11)
smoked for 10–30 years	1,789,335	1,772	7,959,831.9	2.23	0.85(0.81,0.89)	1.04(0.98,1.10)	1.04(0.99,1.11)
smoked for ≥30 years	545,052	596	2,374,378.6	2.51	0.96(0.88,1.04)	0.99(0.90,1.07)	0.98(0.90,1.07)
Current smoker							
smokes for <10 years	891,176	521	3,778,089.1	1.38	0.525(0.48,0.57)	0.77(0.71,0.85)	0.791(0.72,0.87)
smokes for 10–30 years	3,424,942	1,868	15,042,614.6	1.24	0.47(0.45,0.50)	0.65 (0.62,0.69)	0.67(0.63,0.70)
smokes for ≥30 years	1,287,381	904	5,553,769.4	1.63	0.62(0.58,0.66)	0.67(0.63,0.72)	0.68(0.63,0.73)

Model 1 Not adjusted.

Model 2 Adjusted by age and sex.

Model 3 Adjusted by age, sex, regular exercise, drinking status, BMI, diabetes mellitus, hypertension, dyslipidemia, history of stroke, and history of myocardial infarction

## Discussion

We report a negative association between tobacco smoking and newly diagnosed vitiligo using a nation-wide cohort database. No study has reported on the incidence of vitiligo associated with smoking status.

The detrimental effects of smoking have been reported for several skin diseases. Palmoplantar pustulosis is well known to be prevalent in current or ex-smokers^20^. Psoriasis is related to smoking habits and heavier smoking is reported to increase the relative risk of psoriasis and its severity^16–19^. Smoking is also considered a triggering factor in hidradenitis suppurativa^21–27^ and systemic lupus erythematosus^37–39^. Smoking has detrimental effects on wound healing^40^ and skin aging^41–43^.

In contrast, protective effects of smoking have also been reported for several skin diseases. Case-control studies report that pemphigus vulgaris and foliaceous occur less frequently in current and ex-smokers^28–33^. Development of aphthous ulcers is associated with cessation of smoking^44–50^. The beneficial effects of smoking on oral ulcers in Behçet’s disease has been shown in several reports^34–36^. Behçet’s disease oral lesions appear after cessation of smoking; therefore, the beneficial effects of nicotine have been suggested^51–54^. However, some studies report that smoking is not a significant risk factor for Behçet’s disease^55^, and smoking is a risk factor for vasculitis and neurological manifestations of the disease^56,57^. (Table 4) In addition to skin diseases, Parkinson’s disease^58–60^ is inversely associated with smoking without exact known mechanisms. Ulcerative colitis is well-known to be inversely associated with smoking^61^. Until now, the association between vitiligo and smoking has not been evaluated or reported.

**Table 4:** Effects of smoking on skin disease.

Detrimental	Protective
Palmoplantar pustulosis^20^	Pemphigus vulgaris^28–33^
Psoriasis^16–19^	Aphthous ulcer^44–50^
Hidradenitis suppurativa^21–27^	Oral ulcer in Behçet’s disease^34,35,51–54^
Systemic lupus erythematosus^37–39^	
Wound healing^40^	
Skin aging^41–43^	

The pro-oxidant and anti-oxidant effects of smoking are suggested in several studies. Tobacco contains a variety of reactive oxygen species (ROS). Therefore, smoking has deleterious effects on human health. Otherwise, tobacco also contains compounds that inhibit the activity of monoamine oxidase (MAO) and can reduce the level of ROS produced by MAO. There has been a report that smokers have lower MAO activity than never-smokers^62,63^. Another study reported that nicotine chelates ferrous ions that produce oxygen radicals and functions as an antioxidant^64^.

In this study, the risk of vitiligo decreased in current smokers in relation to smoking dose. This result was counter to our expectation that the oxidative stress incurred from smoking would cause vitiligo development. Vitiligo is an acquired depigmentation skin disease caused by destruction of melanocytes in affected skin. Oxidative stress and autoimmunity are important in pathogenic events in melanocyte loss^65^. Increased levels of ROS, mainly H_2_O_2_, are reported in the epidermis of vitiligo skin^66^. Elevated NADPH oxidase activity and increased production of ROS and reactive nitrogen species are reported in vitiligo patients^67^. Increased activity of MAO is reported in vitiligo epidermis that results in accumulation of H_2_O_2_^68^. Low levels of antioxidants, such as catalase, glutathione peroxidase, and superoxide dismutase are reported in the epidermis and serum of vitiligo patients^69,70^. The inverse relationship between vitiligo and smoking might be explained by inhibitory effects on MAO due to tobacco smoking^62^. However, the exact mechanism needs to be evaluated by further studies.

Considering the harm caused by smoking and the lack of a distinct benefit for vitiligo development, we do not think smoking should be considered as a prevention or treatment option for vitiligo. We suggest investigating the mechanism of smoking in vitiligo development *in vitro* and *in vivo* in further studies. We present a new topic in vitiligo research in a field with little progress and limited development of treatment options.

There are several limitations in our study. First, the genetic susceptibility of individuals or family history of vitiligo was not evaluated. Second, causal links between smoking and vitiligo could not be identified in an epidemiologic study. Third, the status of smoking was obtained from self-reported questionnaires during the health examination and a response bias caused by respondents failing to provide correct information cannot be ruled out. And the absence of data on time since quitting smoking in ex-smokers is considered as a limitation in this study. The role of smoking in vitiligo development remains for additional clinical analysis or *in vitro* studies.

Several strengths in this study are that first, we examined the relationship between vitiligo and smoking in the South Korean population. This study used a nation-wide population-based cohort. In Korea, a general health examination, either biannually or annually according to occupation, is mandatory for local household owners, office employees, and family members over the age of 40 years. The total number of individuals who underwent National Health Examinations in Korea from 2009 until 2012 was 23,503,802 that was more than half of the total population of Korea. Considering that we excluded subjects less than 20 years old, the results of this study could generalize the majority of Koreans who receive a medical examination. We identified the possible suppressive effects of smoking on the development of vitiligo. Current smokers showed a decreased risk of vitiligo development compared to non-smokers regardless of age and sex, and the amount of smoking was negatively correlated with vitiligo development. The suppressive mechanism of smoking on the cutaneous autoimmune disease, vitiligo, is unknown. Further *in vitro* studies are needed to investigate the exact mechanism between smoking and vitiligo. However, this study helps to broaden the understanding of vitiligo pathogenesis.
